# Anterior Chest Wall Pilonidal Sinus Mimicking Recurrent Abscess: Diagnostic Pitfalls and Therapeutic Implications

**DOI:** 10.3390/diagnostics16152481

**Published:** 2026-08-06

**Authors:** Di Xu, Yimin He, Fangyi Wu, Jun Fu, Ying Gao

**Affiliations:** 1 Department of Thoracic Surgery, The Central Hospital of Wuhan, Tongji Medical College, Huazhong University of Science and Technology, Wuhan 430014, China; xudi@zxhospital.com; 2 Department of Dermatology, The Central Hospital of Wuhan, Tongji Medical College, Huazhong University of Science and Technology, Wuhan 430014, China; wendyhym@126.com (Y.H.); 18062761256@163.com (F.W.)

**Keywords:** pilonidal sinus, chest wall, sinus tract

## Abstract

Pilonidal sinus is a chronic inflammatory skin condition predominantly found in the sacrococcygeal region, characterized by recurrent infections and sinus tract formation. Occurrences outside this area, particularly on the anterior chest wall, are exceedingly rare and prone to misdiagnosis. We report a rare case of a 24-year-old Chinese man presenting with a 3-year history of a recurrent, draining lesion on the anterior chest wall. Initial treatment via simple incision and drainage for a presumed sebaceous cyst resulted in delayed wound healing and persistent purulent discharge. Subsequent magnetic resonance imaging (MRI) and computed tomography (CT) revealed a localized superficial lesion. Definitive surgical debridement uncovered a sinus tract containing embedded hair fragments. Histopathological examination confirmed a pilonidal sinus with a robust foreign body giant cell reaction and chronic inflammation. After complete resection, during the nearly two-year follow-up period, the healing has been smooth, and no recurrence has been observed. This case highlights the necessity of detailed clinical history-taking and the inclusion of ectopic pilonidal sinus in the differential diagnosis of refractory chest wall abscesses to prevent repeated, ineffective interventions.

**Figure 1:**
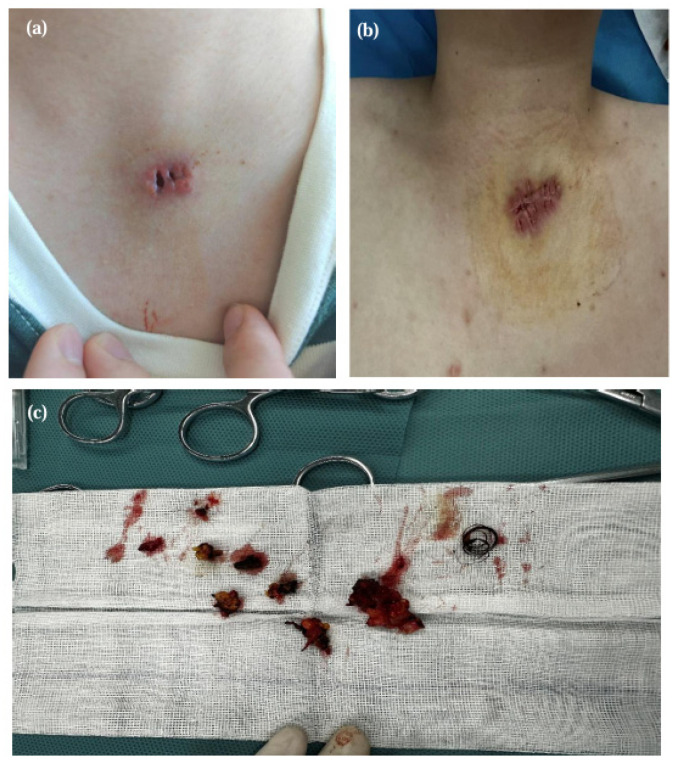
A 24-year-old man presented with a three-year history of a chest wall abscess and recurrent cutaneous ulceration. On 21 June 2024, he underwent a cyst removal surgery in the Department of Dermatology at our institution, and histopathological examination revealed a sebaceous cyst. Despite the initial surgery, the wound failed to achieve complete healing, with intermittent purulent drainage and associated erythema and tenderness (**a**). Two months after the initial surgery, he presented to our Department of Thoracic Surgery for further evaluation and underwent chest wall debridement and closure (**b**). During the surgery, extensive necrotic tissue was debrided, revealing a sinus tract and hair shafts within the chest wall subcutis (**c**). During this period, we re-examined the pathological specimens from the first surgery and found that the initial surgical removal was incomplete, and there were no characteristic hair fragments.

**Figure 2:**
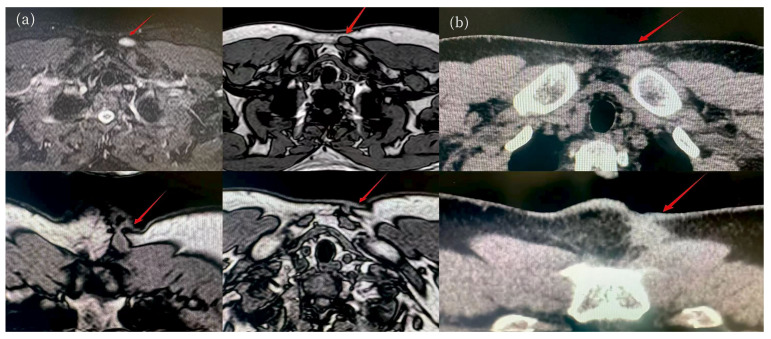
Pilonidal sinus refers to the foreign body reaction elicited by hair embedded within surrounding tissues, most commonly occurring in the sacrococcygeal region, whereas ectopic manifestations on the trunk are rare [1,2,3]. During the acute phase, symptoms typically include redness, swelling, warmth, and pain, with or without purulent drainage. Complications often include difficult-to-heal wounds and the generation of sinus tracts. The clinical manifestations of chronic pilonidal disease vary based on the inflammatory response and skin scarring, with deeper lesions communicating with the skin surface via sinus tracts, allowing for fluid discharge under pressure [4]. We report a case of chest wall pilonidal sinus, highlighting the importance of careful history-taking and considering ectopic pilonidal sinus in the differential diagnosis of refractory chest wall abscesses, so as to avoid misdiagnosis, repeated interventions, and recurrence. Magnetic resonance imaging (MRI) revealed a relatively homogeneous lesion with prolonged T1 and prolonged T2 signal intensities; a cutaneous sinus tract was observed in the anterosuperior aspect of the lesion (**a**). Computed tomography (CT) demonstrated a well-defined, oval, iso-attenuating nodule measuring 2.0 × 1.0 cm in cross-section, located in the deep subcutaneous fat layer of the anterior chest wall (**b**), immediately adjacent to the origin of the left sternocleidomastoid muscle and the anterior margin of the subclavius muscle, with preservation of the intervening fascial plane. Pilonidal cysts can occur at virtually any site on the body, yet the sacrococcygeal region and the upper part of the gluteal cleft remain the most common locations. The present case, however, describes an ectopic pilonidal sinus arising from the chest wall that may be related to local repeated friction and minor trauma. Nevertheless, this ectopic entity shares the general clinical features of ordinary sacrococcygeal pilonidal sinus. In the acute phase, it presents with pain, tenderness, and swelling, resembling a superficial abscess elsewhere in the body. Because the lesion is not completely excised, relying solely on local debridement and drainage fails to achieve radical cure, and the disease gradually progresses to the chronic phase. This is characterized by persistent chronic inflammatory changes in the skin and wound nonhealing, with the deep-seated lesion communicating with the skin surface through the sinus tract, from which fluid may exude under pressure. Therefore, whenever a superficial mass recurs or the wound repeatedly fails to heal after excision, particularly if foreign materials such as hairs are discovered in the wound, the possibility of pilonidal sinus must be considered, regardless of the anatomical location. For diagnostic imaging, CT and MRI are distinctly superior to color Doppler ultrasonography, as they are more effective in delineating the underlying sinus tract. Once the diagnosis of pilonidal sinus is established, prompt treatment is warranted; however, complete surgical excision remains the only definitive curative approach. In our case, the patient underwent total excision of the lesion, and the postoperative course was uneventful. The wound healed well, and sutures were successfully removed on the seventh postoperative day. The patient was followed up a month post-surgery, with satisfactory wound healing, and there has been no recurrence to date.

**Figure 3:**
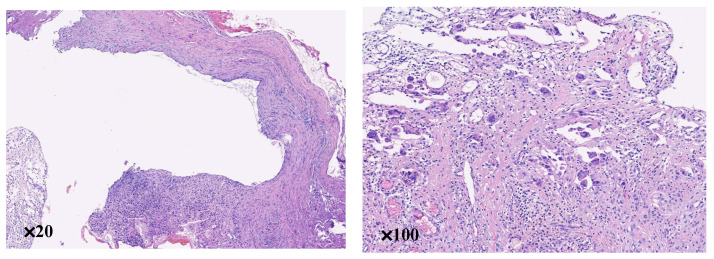
Pathological analysis of the tissue revealed chronic inflammatory cell infiltration, no epithelial structure was found after consecutive sections were made, and immunohistochemistry showed CD34 and beta-catenin negativity. A drain was inserted to manage any fluid accumulation, but minimal output was observed, allowing for its removal on the second postoperative day. We need to rule out several differential diagnoses for a refractory draining anterior chest wall lesion: epidermal inclusion cyst/sebaceous cyst, foreign body granuloma, chronic abscess and cutaneous fistula, which have no evidence of hair, but calcification or enhancement can be observed on the image. In addition, differential diagnosis includes dermatofibrosarcoma protuberans (DFSP), solitary fibrous tumor (SFT) and desmoid fibromatosis [5]. DFSP manifests as an infiltrative subcutaneous mass prone to mimic pilonidal sinus after ulceration. Microscopically, spindle cells grow in a storiform pattern with diffuse CD34 positivity and negative β-catenin. Pilonidal sinus consists of inflammatory granulation and reactive fibrous tissue, negative for both markers. SFT, originally discovered in pleura but also seen in skin, presents as a painless subcutaneous lesion that may ulcerate. It shows alternating hyper/hypocellular zones and characteristic staghorn vessels, with diffuse CD34 and absent β-catenin staining. Desmoid fibromatosis is a slow-growing, recurrent mass with occasional pain. Plump spindle cells lie in myxocollagenous stroma with peripheral inflammatory infiltrates; tumor cells are CD34-negative and show nuclear β-catenin positivity. Delay in diagnosis may lead to prolonged wound healing, repeated drainage procedures, delayed definitive treatment.Ultimately, we diagnosed pilonidal sinus of the chest wall. Three main factors contribute to the development of pilonidal sinus: penetration of shed hair into the skin causing irritation, the force of hair implantation, and a decrease in skin resistance due to hair invasion [6]. Surgical excision remains the definitive treatment for pilonidal sinus [7]. However, simple incision and drainage fail to address the underlying factors, resulting in a high rate of recurrence postoperatively [8]. In acute abscesses, drainage may be appropriate initially, but definitive management requires identification and excision of the sinus tract and removal of hair/foreign material [9].

## Data Availability

The raw data supporting the conclusions of this article will be made available by the authors on request.

## Abbreviations

The following abbreviations are used in this manuscript:

CT	computed tomography
MRI	magnetic resonance imaging
